# *In Vitro* Antiplasmodial, Cytotoxicity, and Antioxidant Activities of *Lophira lanceolata* (Ochnaceae): A Cameroonian Plant Commonly Used to Treat Malaria

**DOI:** 10.1155/2023/4061592

**Published:** 2023-02-11

**Authors:** Mounvera Abdel Azizi, Noumedem Anangmo Christelle Nadia, Yamssi Cedric, Gamago Nkadeu Guy-Armand, Ngouyamsa Nsapkain Aboubakar Sidiki, Tientcheu Noutong Jemimah Sandra, Tako Djimefo Alex Kevin, Vincent Khan Payne

**Affiliations:** ^1^Department of Animal Biology, Faculty of Science, University of Dschang, P.O. Box 067, Dschang, Cameroon; ^2^Department of Microbiology, Hematology and Immunology Faculty of Medicine and Pharmaceutical Sciences, University of Dschang, P.O. Box 96, Dschang, Cameroon; ^3^Department of Biomedical Sciences, Faculty of Health Sciences, University of Bamenda, P.O. Box 39, Bambili, Cameroon; ^4^Department of Animal Organisms, Faculty of Science, University of Douala, P.O. Box 24157, Douala, Cameroon

## Abstract

**Background:**

Malaria is the leading cause of morbidity and mortality in African countries. We aimed this study at evaluating the *in vitro* antiplasmodial, antioxidant, and cytotoxicity activity of *Lophira lanceolata* extracts.

**Method:**

The aqueous and ethanol extracts were obtained by maceration. It tested *in vitro* the extracts against *Plasmodium falciparum* 3D7 and multiresistance Dd2. Macrophage cell lines (RAW 264.7 cells) and red blood cells were used for cytotoxicity tests. The antioxidant activity was assessed by 1,1-diphenyl-2-picrylhydrazine (DPPH), hydrogen peroxide (H_2_O_2_), nitric oxide (NO) reduction, and ferric reducing antioxidant power (FRAP) scavenging.

**Results:**

The *in vitro* antiplasmodial results showed that the ethanol extract was the most active, with IC50 of 24.51 ± 4.77 *µ*g/mL and 31.86 ± 3.10 *µ*g/mL, respectively, on the resistant Dd2 and sensitive 3D7 strains unlike the aqueous which indicated moderate activity with an IC_50_ of 51.36 ± 4.86 *μ*g/mL and 56.36 ± 4.27 *μ*g/mL, respectively, on the resistant Dd2 and sensitive (3D7) strains. However, the ethanol extract had the highest activity, with an IC_50_ of 8.153 g/mL, 1915 g/mL, 30.81 g/mL, and 54.66 g/mL, respectively, for DPPH, H_2_O_2_, NO, and FRAP, while the aqueous extract had an IC_50_ of 6.724, 2387681, 185.7, and 152.0 g/mL, respectively, for DPPH, H_2_O_2_, NO, and FRAP. The cytotoxicity test reveals that both extracts do not promote red blood cell haemolysis. They presented weak activity against RAW 264.7 cells and red blood cells.

**Conclusion:**

According to these findings, the aqueous and ethanol extracts have antiplasmodial and antioxidant activity but with no cytotoxic effects on red blood cells or RAW cells. However, it will be important to investigate the *in vivo* antiplasmodial and antioxidant activity of these extracts.

## 1. Background

Malaria is a parasitic disease that affects people all over the world. According to the World Malaria Control Report [1], there were 228 million malaria cases and 405,000 deaths in 2018, while in 2017, 231 million cases and 416,000 deaths. Malaria is the leading cause of morbidity and mortality in tropical countries, affecting pregnant women and children under the age of five [2]. This disease is endemic in tropical regions. The number of cases increased from 224 million in 2015 to 241 million in 2020.

In terms of the number of infected cases and deaths, the African continent bears the lion's share of the global burden. Thus, this continent accounts for over 95% of all malaria cases and 96% of all deaths worldwide [3]. This accounts for 71% of all cases and 85.7% of all deaths worldwide. Every year, about 300 million people in this area of over 400 million people are infected, while 120 million become ill [4].

Malaria is the leading cause of morbidity and mortality in Cameroon's most vulnerable populations. The World Health Organization (WHO) estimates 6,228,154 cases in 2018, and the country is one of the 11 countries with the highest disease burden in the world [1]. The most difficult challenge in the fight against malaria today is the resistance of the parasite to the most common antimalarial drugs. Faced with this difficult problem, it made constant efforts to develop new effective and inexpensive antimalarial drugs.

There are numerous compounds with a wide range of biological and pharmacological properties found in medicinal plants [5]. They have long been used to treat a variety of illnesses in humans and other living organisms, and their usage has always been practical [6]. In addition to the typical medicinal plants, which are frequently being used, much work needs to be performed in order to compile an exhaustive list of the species that are likely to have antiplasmodial therapeutic uses. Noumedem et al. [7] demonstrated that there is a close relationship between malaria and oxidative stress. According to these authors, when the Plasmodium parasite infects an organism, it stimulates an overproduction of free radicals. These free radicals are not only toxic to the parasite but also equally toxic to the host organism. Hence, it will be of paramount importance to have drugs that will have both antiplasmodial and antioxidant properties.

*Lophira lanceolata* is a useful tree in Cameroon whose seeds are eaten. It is known that we can use every part of the plant for pest control, such as food, medicine, and mystical practices. In conventional medicine, the oil is applied to the skin to avoid dryness and is used to treat dermatitis, toothache, and muscle exhaustion [8]. Children are given the oil as a tonic after mixing it with oatmeal. Women are advised to consume a decoction made from the roots and fresh or dried young leaves to combat menstrual pain, digestive ailments, diarrhoea, dysentery, and malaria. In the West Region of Cameroon, the bark is also used to cure fevers, bacterial infections, and digestive issues. The bark of the root is a treatment for yellow fever, and they used an infusion made from the bark and leaves as an antitrypanosomal drug. The aim of this study is to provide scientific evidence on the use of *Lophira lanceolata* as an antiplasmodial remedy used by traditional healers to treat malaria to justify its usage.

## 2. Material and Method

### 2.1. Plant Material Collection and Authentication

The stem bark, flowers, seeds, and leaves of *L. lanceolata* were harvested in Foumbot Subdivision Noun Division, West Region of Cameroon. The National Herbarium identified this plant with voucher number 4002B.

### 2.2. Preparation of Aqueous and Ethanol Extracts

We used ethanol to make the extraction because during the survey the traditional practitioner told us that they use fermented palm wine (ethanol) or infusion to prepare this remedy.

The ethanol extract was prepared according to the method described by Josué [9]. Briefly, 50 g of *L. lanceolata* plant material was macerated in one liter of ethanol for three days (the duration of 72 h is to maximize the extraction of the compounds) at room temperature in a tightly closed jar, and the mixture was stirred regularly. The mixture obtained was filtered using a sieve and Wattman number 1 filter paper. The filtrate obtained was then evaporated in an oven at 40°C until a dry residue was obtained.

For the aqueous extract, 50 g of *L. lanceolata* stem is introduced as bark powder in one liter of hot distilled water (100°C), and then the whole mixture is left to cool down for some minutes. After cooling, the mixture was filtered using filter paper, and the filtrates obtained were sieved and filtered using Wattman filter paper number 1 and dried in an oven at 50°C (above 50°C the active ingredient will be degraded) to obtain a dry residue.

### 2.3. *In Vitro* Evaluation of Antiplasmodial Activity

#### 2.3.1. *Plasmodium falciparum* Strain Culture

*Plasmodium falciparum* strains were cultured using the Trager and Jensen method [10] with slight modifications. In brief, fresh human red blood cells (RBCs) from group O^+^ individuals were used to culture the chloroquine-sensitive*Plasmodium falciparum* strain 3D7 and the multiresistant Dd2 in complete Roswell Park Memorial Institute medium (RPMI 1640) (500 mL RPMI 1640 (Gibco, UK) supplemented with 25 mM HEPES (Gibco, UK), 0.50% Albumax I (Gibco, USA), 1X hypoxanthine (Gibco, USA), and 20 *µ*g/mL gentamicin (Gibco, China)) and incubated at 37°C in a humidified incubator consisting of 92% N_2_, 5% CO_2,_ and 3% O_2_.

#### 2.3.2. Synchronization of Cultures

Parasite cultures containing mostly ring stages (>80%) were synchronized at the same evolutionary stage (ring stage) using D-sorbitol before each antiplasmodial activity test.

#### 2.3.3. *In Vitro* Antiplasmodial Activity Using SYBR Green (an Asymmetrical Cyanine Dye Used as a Nucleic Acid Stain) Fluorescence

The *in vitro* antiplasmodial activity was carried out according to the method described by Akala et al. [11]. In brief, 190 *µ*L of a parasite suspension at 2% parasitemia and 1% hematocrit was introduced into a 96 microtitre plate with 10 *µ*L of different plant extract concentrations. We used artemisinin and chloroquine as the positive control while 1% dimethylsulfoxyde (DMSO) was used as the negative control. We incubated the microtitre plate at 37°C in a CO_2_ incubator (92% N_2,_ 5% CO_2,_ and 3% O_2_) for 72 h. The final concentration in the microtitre plate ranged from 200 to 0.0128 *µ*g/ml for the extract and from 1 to 0.000064 *µ*m for artemisinin and chloroquine in a final volume of 100 *µ*L. After 72 h of incubation, 100 *µ*L of SYBR Green was added to each well followed by 1h of incubation at 37°C in darkness. The fluorescence was measured using a Tecan infinite M200 microplate reader at an excitation and emission wavelength of 485 and 538 nm, respectively.

We calculated the resistance index using the following formula: resistance index (RI) = IC_50_ extract on PfDd2/IC_50_ of the same extract on 3D7.

### 2.4. Cytotoxicity Test

#### 2.4.1. Haemolysis Test

The method described by Sinha et al. [12] was used. Briefly, 500 *µ*L of a suspension of erythrocytes from blood group O^+^ at 4% of heamatocrit was prepared in incomplete RPMI 1640 in the presence of 500 *µ*l of the extract at various concentrations in Eppendorf tubes with a final concentration of the extracts ranging from 1000 to 625 g/mL. Triton X-100 at 0.5% in a final volume of 1000 *µ*L was used as the positive control while erythrocyte suspension in incomplete culture media at 4% was used as the control. The plates were incubated for 3 h at 37°C in a CO_2_ incubator. We measured the absorbance at 540 nm using a Tecan Infinite M200 microplate reader. We calculated the haemolysis rate of the different extracts by using the following formula:

(1) $$\begin{array}{c} percentage\;of\;haemolysis\left(\%\right)=\frac{\left(OD\;extract-OD\;negative\;control\right)}{OD\;positive\;control}*100\text{.} \end{array}$$

#### 2.4.2. Cytotoxicity Test on RAW 264.7Cell

We evaluated this test using the resazurin-based assay described by Balbaie et al. [13]. Macrophages were seeded in a 96-well microtitre plate titer at a density of 10^4^ cells in 100 *µ*L of complete medium and incubated for 24 h at 37°C in a CO_2_ incubator. The extracts at various concentrations were added to each well and incubated for another 48 h, and cell proliferation was evaluated by the addition of 10 *µ*L of 0.15 *µ*g/mL of resazurin into each well followed by 4 h incubation. It measured the absorbance at 540 nm using the Tecan infinite M200 plate reader, and it expressed the results as 50% cytotoxic concentration. The selectivity index (SI) was calculated as follows:

(2) $$\begin{array}{c} SI=\frac{CC50\;of\;RAW\;cell}{IC50\;Of\;plasmodium}\text{.} \end{array}$$

### 2.5. Antioxidant Activity

#### 2.5.1. 2, 2-Diphenyl-1-Picryl Hydrazyl (DPPH) Radical Scavenging Test

The method described by McCune and John [14] was used to evaluate this test. Briefly, 4 mg of DPPH was dissolved in 100 ml of methanol to produce a methanol solution of DPPH. Nine hundred and fifty microlitter of the DPPH solution was added to 50 *µ*L of extract. We evaluated the extracts at various concentrations (1, 3, 10, and 30), along with the reference drug (ascorbic acid). It expressed a radical scavenging effect as percentage inhibition (PI) using the following formula:

(3) $$\begin{array}{c} IP=\frac{\left(AO-A1\right)}{AO}*100\text{.} \end{array}$$

A0 (DPPH absorbance), A1 (sample absorbance), and IC_50_ (concentration of the sample necessary to neutralize 50% of the free radicals) were obtained.

#### 2.5.2. Hydrogen Peroxide Trapping

The capacity of plant extracts to break down hydrogen peroxide was measured using the Özyürek et al. [15] technique. At room temperature, 0.4 mL of the extract at various concentrations was immediately added to 0.6 mL solution of hydrogen peroxide (40 mM) produced in phosphate buffer (pH 7.4; 50 mM) and was incubated for 10 minutes. We measured the absorbance of the combination at 230 nm using a spectrophotometer. We used ascorbic acid as a positive control.

#### 2.5.3. Nitric Oxide Production Inhibition Test

We carried out this test according to the method described by Ashokkumar et al. [16] with slight modifications. In brief, 1520 *µ*L of sodium nitroprusside (10 mM) was added to test tubes containing 180 *µ*L of *L. lanceolata* extract or vitamin C. The mixture was incubated for 2 hours and 30 minutes at 25°C. After incubation, an equivalent volume of 1% sulfanilamide was collected and placed and 500 *µ*L of the preceding combination in a cuvette, followed. The mixture was homogenized and incubated at room temperature for 5 minutes in darkness. It measured the optical density of the produced chromatophore at 530 nm within 30 minutes. The following formula was used to obtain the inhibition percentages:

(4) $$\begin{array}{c} \%\;inhibition=\frac{ODcontrol-ODsample}{ODcontrol}x100\text{.} \end{array}$$

#### 2.5.4. Evaluation of the Reducing Power of the Extracts

We evaluated the reducing power according to the method described by Athukorola et al. [17]. In brief, 0.2 ml of increasing concentration of *L. lanceolata* extract was added in the test tubes, followed by the addition of 0.5 mL of phosphate buffer (200 mM, pH 6.6) and 0.5 mL of potassium ferricyanide (30 mM). We incubated the whole mixture at 50°C for 20 minutes. Subsequently, 0.5 mL of trichloroacetic acid solution (6 M) was added and we centrifuged the mixture for 10 minutes at 3000 rpm. A 0.5 mL of the supernatant was introduced followed by 0.5 mL of distilled water and 0.1 mL of iron III chlorides (FeCl_3_) at a concentration of 6 mM and was incubated for 10 minutes at 37°C. It measured the optical densities at 700 nm while it used vitamin C as a reference substance.

### 2.6. Qualitative Phytochemical Screening

The extracts were tested for the presence of sterols, alkaloids, triterpenoids, saponins, anthocyanins, and anthraquinones using standard procedures described by Harbone [18].

### 2.7. Total Phenolic and Flavonoid Contents

The total Phenolic content was determined using the method described by Singleton and Rossi [19] while the flavonoid content was determined using the method described by Djeussi et al. [20].

### 2.8. Statistical Analysis

The fluorescence values obtained were used to calculate the percentage inhibition using Microsoft Excel software. Subsequently, the 50% inhibitory concentrations (IC50) were determined using the concentration-response curves obtained by plotting the logarithm of the concentration as a function of the percentage inhibition using GraphPad Prism 8 software.

## 3. Results

### 3.1. *In Vitro* Antiplasmodial Activity

Table 1 shows the *in vitro* antiplasmodial activity. The table suggests that the aqueous extract has little effect on PfDd2 and Pf3D7 while the ethanol extract was active against both strains.

### 3.2. Cytotoxicity

#### 3.2.1. Haemolysis Test

**Figure 1:**
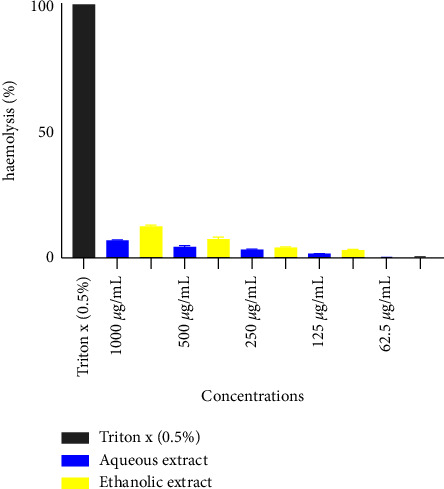
Effect of *L. lanceolata* on haemolysis.

Figure 1 shows the effect of *L. lanceolata* on haemolysis. It appears from the figure that the haemolysis rate of aqueous and ethanol extracts is significantly lower (at all test concentrations) than triton (0.5%) which is the negative control.

#### 3.2.2. Cytotoxicity on RAW 264.7 Cells

The effect of *L. lanceolata* on RAW 264.7 cells is presented in Table 2. It follows from the analysis of the table that CC_50_ of the aqueous extract was >1000 *µ*g/mL while that of the ethanol extract was 622.45 *µ*g/mL. The aqueous extract had a SI of 19.53 and 17.74 *µ*g/mL for the PfDd2 and Pf3D7 strains, while that of ethanol was 19.53 and 25.39 *µ*g/mL for the Pf3D7 and PfDd2 strain, respectively. This index indicates an activity against the *Plasmodium falciparum* strain.

### 3.3. *In Vitro* Antioxidant Activity

#### 3.3.1. DPPH Radical Scavenging Activity

**Figure 2:**
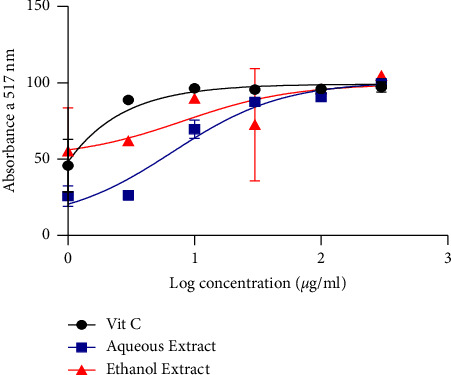
DPPH radical scavenging activity.

Figure 2 demonstrates the DPPH scavenging activity of *L. lanceolata*. It appears from the figure that the aqueous and ethanol extracts had scavenging activity with IC_50_ of 6.724 *µ*g/mL and 8.153 *µ*g/mL, respectively. Ascorbic acid, which served as the positive control, presented a significantly low IC_50_ value indicating its high scavenging activity.

#### 3.3.2. Hydrogen Peroxide Scavenging Activity

**Figure 3:**
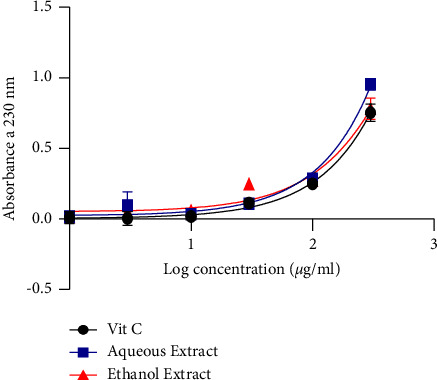
H_2_O_2_ radical scavenging activity.

Figure 3 shows the hydrogen peroxide scavenging activity. It appears from the figure that the aqueous extract inhibited the production of H_2_O_2_ more than ethanol and ascorbic acid with an IC_50_ of 1915 *µ*g/mL.

#### 3.3.3. Nitric Oxide (NO) Scavenging Assay

**Figure 4:**
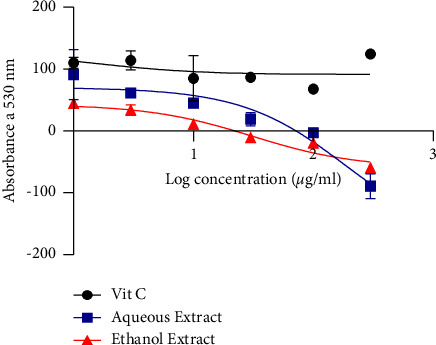
NO radical scavenging activity.

The scavenging activity of NO is presented in Figure 4 in terms of PI. The aqueous extract revealed the highest scavenging activity compared to the ethanol extract, with IC_50_ of 185.8 *µ*g/mL and 30.18 *µ*g/mL, respectively.

#### 3.3.4. Ferric Reducing/Antioxidant Power of *L. lanceolata*

**Figure 5:**
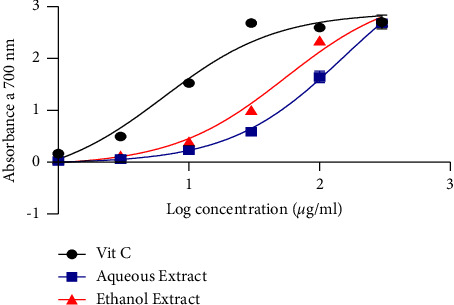
Ferric-reducing power of *L. lanceolata*.

Figure 5 shows the ferric reducing power of *L. lanceolata*. It follows from the analysis of the figure that the aqueous extract presented the lowest ferric-reducing power while ascorbic acid presented the highest ferric-reducing power.

### 3.4. Qualitative Phytochemical Screening

Table 3 shows the phytochemical screening of the aqueous and ethanol extracts of *L. lanceolata* stem bark. It follows from the analysis of the table that the aqueous extract contains alkaloids, saponins, triterpenoids, anthocyanins, and anthraquinones. Similarly, the ethanolic extract contains the same compounds except for saponins.

### 3.5. Total Phenolic and Flavonoid Contents

**Figure 6:**
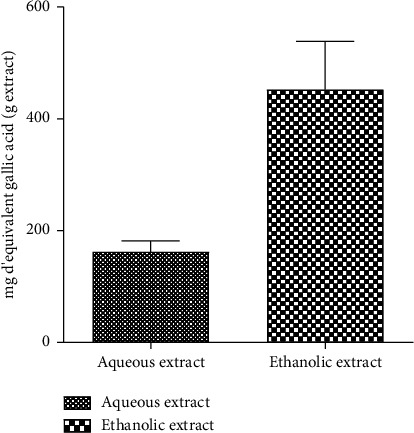
Total flavonoid content of the aqueous and ethanol extracts.

**Figure 7:**
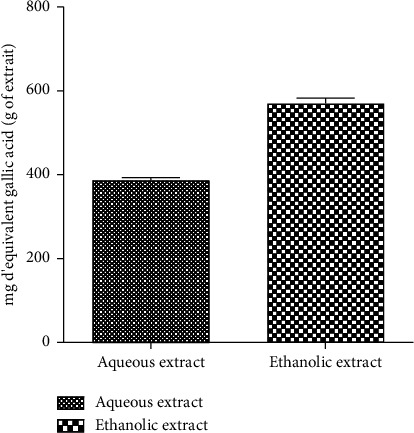
Total phenolic content of the aqueous and ethanol extracts.

Figure 6 shows the amount of flavonoid present in each extract. It appears from the figure that the ethanol extract (452.2 ± 86.66 mg/g) contains more flavonoids than the aqueous extract (162.2 ± 19.68 mg/g).  Figure 7 shows the total phenolic content of the aqueous and ethanol extracts. Similarly, more phenolic compounds were found in the ethanol extract (569.7 ± 12.82 mg/g) compared to the aqueous extract (386.8 ± 6.235 mg/g).

## 4. Discussion

The stem bark of *Lophira lanceolata* is used to treat fever and malaria in the Western Region of Cameroon by traditional healers. These extracts were examined for their *in vitro* antiplasmodial activity on chloroquine-sensitive (3D7) and chloroquine-resistant (Dd2) strains, and the result showed that the *in vitro* antiplasmodial test was dose-dependent with IC_50_ of 56.365 *µ*g/mL and 51.36 *µ*g/mL for the Pf3D7 and PfDd2 for the aqueous extract. Concerning the ethanol extract, the IC_50_ observed was 31.865 *µ*g/mL and 24.515 µg/mL for the Pf3D7 and the resistant PfDd2. According to Njagi et al. [21], an extract is regarded to be very active if IC_50_ < 5 *µ*g/mL, active if 5  <  IC_50_ < 50 *µ*g/mL, weakly active if 50 *µ*g/mL < IC_50_ < 100 *µ*g/mL, and inactive if IC_50_ > 100 *µ*g/mL. Based on this classification, the ethanol extract was active compared to the aqueous extract which was weakly active. The results obtained with the ethanol extract agree with the study conducted in Nigeria by Falade et al. [22] where they carried out an antiplasmodial activity on *P. falciparum* NF54 (sensitive) and K1 (multiresistant) strains of methanol and ethyl acetate extracts of *Lophira alata* and obtained an IC_50_ of 11.3 ± 3.6 *μ*g/mL (NF54) and 5.3 ± 3.6 *μ*g/mL (K1) and 9.7 ± 2.7 *μ*g/mL (NF54) and 59.4 ± 1.4 *μ*g/mL (K1), respectively. These results are in contradiction with those of Falade et al. [22] where they had an IC_50_ of 2.5 ± 0.3 *μ*g/mL and 2.5 ± 2.1 *μ*g/mL on NF54 and K1strain when using the hexane extract of *Lophira alata*. Furthermore, the study of Ajaiyeoba et al. [23] on K1 resistant strain obtained an IC_50_ of 156.6 ± 5.70, which contradicts with the IC_50_ of the aqueous and ethanol extracts reported in our study. The RI of aqueous and ethanol extracts was reported to be 0.91 and 0.01, respectively. The differences observed in these results could be explained by several parameters such as the Plasmodium strains used, the solvent used for extraction, the method of extraction, the chemical composition of the plant, the geographical location of the plant, and the period of harvesting the plant. The antiplasmodial activity may be observed in this study because of the phytochemical constituents of the plant that might have acted by destroying the parasite cytosol, membrane, mitochondria, and digestive vacuole of the parasites. Moreover, the elimination of haemozoin biocrystallization by alkaloids, control of *P. falciparum* fatty acid biosynthesis, and disruption of protein synthesis by triterpenoids are other potential mechanisms of parasite inhibition by plant extracts.

The results of the cytotoxicity study showed that the stem bark extracts of *L. lanceolata* were nontoxic to human cells. According to the American National Cancer Institute (ANCI), the threshold for crude extract toxicity is a CC_50_ of less than 30 *µ*g/ml [24]. According to these guidelines, CC_50_ ≥ 20 is considered safe against normal human cells. In the present study, the aqueous and ethanol extracts of *L. lanceolata* registered (CC_50_) values are greater than 1000 *µ*g/ml and 622.45 *µ*g, respectively, against the RAW 264.7 macrophage cells. The result obtained is similar to that observed by Falade et al. [22] who obtained an IC_50_ on KB cells greater than 20 *µ*g/ml. An essential criterion for choosing therapeutic plants with high efficacy is the SI activity of the plants against the malaria parasite. It indicates the proportion of biological activity to cytotoxicity. Other studies categorize some medicinal plants' selected indices as low (4 ≤ SI < 10), selective (10 > SI ≤ 25), and extremely selective (≥25). The ethanol extract of *L. lanceolata* indicated a SI of 19 and 53 and 25 and 39, respectively, for Pf3D7 and PfDd2, and the aqueous extract showed SI values of 17 and 74 and 19 and 47 for Pf3D7 and PfDd2, respectively. We, therefore, know if the aqueous and ethanol are active against the malaria parasite based on the above classification. This implies that the extracts contain elements with potential antiplasmodial action. Concerning the cytotoxicity activity on human red blood cells, the extracts of *L. lanceolata* were revealed to be nontoxic to human RBCs compared to the standard cytotoxic drug, Triton x 100 which was found to be highly toxic to human erythrocytes (100%). The percentage of haemolysis induced by the aqueous and ethanol extracts at high concentrations (1000 µg/ml) was 6,99 ± 0,12% and 12,51 ± 0,34%, respectively. Hence, the extracts can be less toxic at lower and higher concentrations [25]. This test was conducted because it is a good indicator of cytotoxicity.

Investigation of the DPPH scavenging capacity showed high IC_50_ of 8.153 *µ*g/mL and 6.724 *µ*g/mL, respectively, for the ethanol and aqueous extracts compared to ascorbic acid with a low IC_50_ of 0.00037 *µ*g/mL. This reaction was dose-dependent. This result corroborates that of Bougandoura and Bendimerad [26], who showed that the hydrogen-donating properties of antioxidant compounds such as ascorbic acid and other molecules decrease and decolorize DPPH. The antioxidant activity is likely caused by these compounds, which are present in both aqueous and ethanol extracts. In the presence of metal ions such as copper or iron, hydrogen peroxide is a significant generator of harmful hydroxyl-free radicals. The IC_50_ activity of the aqueous extract, ethanol extract, and ascorbic acid was ˜2387681, 1915, and ˜9291 *µ*g/mL, respectively, with a dose-dependent activity. According to these findings, the extracts have a remarkable ability to change electrons into reactive free radicals, changing them into more stable, nonreactive species and stopping the chain reaction of free radicals. These outcomes align with what was observed by El-Haci et al. [27]. This is supported by the various IC_50_ values that were obtained.

The aqueous and ethanol extracts rather boosted the production of NO, in contrast to Vitamin C, which inhibited the synthesis of NO. For the two extracts, we measured a high IC_50_ of 185.7 g/mL and 30.81 g/mL. We also observed that when the concentration increases, this activity declines. Regarding the FRAP scavenging activity of the plant extract, the IC_50_ of ascorbic acid, aqueous, and ethanol extract were 6.386, 152.0, and 54.66 *µ*g/mL, respectively. This study demonstrated that iron reduction is dose-dependent since it changes depending on the concentration and ascorbic acid has a substantially higher potential to reduce Fe^3+^ than the aqueous and ethanol extracts. These findings corroborate with those made in Morocco by Ismaili et al. [28], who also showed this dose-dependent relation. The activity observed can be due to the chemical components of the plant. Additionally, Bougandoura and Bendimerad [26] showed how a compound's reducing power might be a useful predictor of its prospective antioxidant action.

## 5. Conclusion

The result shows an antiplasmodial activity observed with the ethanol extract of *L. lanceolata*. Regarding the antioxidant activity of these extracts, both extracts showed high and promising free radical scavenging activity. However, it would be wise to carry out more *in vivo* studies on these extracts, in particular the ethanol extract, to find out these *in vitro* antiplasmodial activities.

## Figures and Tables

**Table 1:** *In vitro* antiplasmodial activity.

IC_50_ ± SD (*µ*g/mL)				(RI) observation
		PfDd2	Pf3D7	
Extracts	Aqueous	51.36 ± 4.86	56.36 ± 4.27	0.91 moderate
	Ethanol	24.51 ± 4.77	31.86 ± 3.10	0.01 active

Positive control	Artemisinin (*µ*M)	0.033 ± 0.00024	0.035 ± 0.00067	0.94
	Chloroquine (*µ*M)	0.527 ± 0.0405	0.300 ± 0.00669	1.75

RI: resistance index.

**Table 2:** Effect of *L. lanceolata* on RAW 264.7 cells.

	CC_50_	SI	
Extract		**Pf3D7**	**PfDd2**
Aqueous	>1000	>17.741	>19.470
Ethanol	622.45	19.533	25.390

SI: selectivity index. PfDd2 : chloroquine-resistant Plasmodium falciparum, Pf3D7: chloroquine-sensitive Plasmodium falciparum.

**Table 3:** Phytochemical screening of *L. lanceolata* extracts.

Extracts	Alkaloids	Sterols	Saponins	Triterpenoids	Anthocyanins	Anthraquinones
Aqueous	+	−	+	+	+	+
Ethanol	+	−	−	+	+	+

## Data Availability

All data generated and analysed are included in this research article.
